# Trajectory of Healthcare Contact Days for Veterans With Advanced Gastrointestinal Malignancy

**DOI:** 10.1093/oncolo/oyad313

**Published:** 2023-11-28

**Authors:** Whitney V Johnson, Quan H Phung, Vishal R Patel, Alexander K Tsai, Nivedita Arora, Mark A Klein, Anders D Westanmo, Anne H Blaes, Arjun Gupta

**Affiliations:** Division of Hematology, Oncology, and Transplantation, Department of Medicine, University of Minnesota, Minneapolis, MN, USA; Division of Hematology, Oncology, and Transplantation, Department of Medicine, University of Minnesota, Minneapolis, MN, USA; Dell Medical School, The University of Texas at Austin, Austin, TX, USA; Division of Hematology, Oncology, and Transplantation, Department of Medicine, University of Minnesota, Minneapolis, MN, USA; Hematology/Oncology Section, Minneapolis Veterans Affairs Healthcare System, Minneapolis, MN, US; Hematology/Oncology Section, Minneapolis Veterans Affairs Healthcare System, Minneapolis, MN, US; Department of Pharmacy, Minneapolis VA Healthcare System, Minneapolis, MN, USA; Division of Hematology, Oncology, and Transplantation, Department of Medicine, University of Minnesota, Minneapolis, MN, USA; Division of Hematology, Oncology, and Transplantation, Department of Medicine, University of Minnesota, Minneapolis, MN, USA

**Keywords:** time toxicity, contact days, gastrointestinal cancer, veterans affairs

## Abstract

How and where patients with advanced cancer facing limited survival spend their time is critical. Healthcare contact days (days with healthcare contact outside the home) offer a patient-centered and practical measure of how much of a person’s life is consumed by healthcare. We retrospectively analyzed contact days among decedent veterans with stage IV gastrointestinal cancer at the Minneapolis Veterans Affairs Healthcare System from 2010 to 2021. Among 468 decedents, the median overall survival was 4 months. Patients spent 1 in 3 days with healthcare contact. Over the course of illness, the percentage of contact days followed a “U-shaped” pattern, with an initial post-diagnosis peak, a lower middle trough, and an eventual rise as patients neared the end-of-life. Contact days varied by clinical factors and by sociodemographics. These data have important implications for improving care delivery, such as through care coordination and communicating expected burdens to and supporting patients and care partners.

## Teaser

Healthcare contact days (days with healthcare contact outside the home) offer a patient-centered and practical measure of how much of a person’s life is consumed by healthcare. This study retrospectively analyzed contact days among decedent veterans with stage IV gastrointestinal cancer. The results have important implications for improving care delivery.

## Background

Patients with stage IV gastrointestinal cancer have healthcare needs related to the underlying cancer, receipt of and recovery from cancer-directed treatment, and management of comorbidities. This care is often essential but can also end up consuming patients’ lives, especially when uncoordinated.^1^ We have previously developed and described a measure of these time burdens, healthcare contact days, which encompasses days with care received outside the patients’ residence.^1^ We have applied contact days as a clinical trial endpoint and as a real-world measure of patient burden.^2-4^

The Veterans Affairs (VA) Hospital is the largest integrated health system in the US. It serves a population with relatively higher comorbidity burden and lower socioeconomic status than a non-VA population.^5^ We sought to characterize the patterns of contact days experienced by decedent veterans with stage IV gastrointestinal cancer.

## Methods

We conducted a retrospective cohort analysis using the VA Clinical Cancer Registry (VACCR) and Corporate Data Warehouse (CDW) to identify decedents aged 18 years or older with stage IV gastrointestinal cancer (gastroesophageal, pancreatic, hepatobiliary, or colorectal) between 2011 and 2021 who received primary oncologic care at the Minneapolis VA Hospital, Minnesota, USA.^6^ Patients could receive additional community care.

We marked each day of follow-up (diagnosis to death) as a contact day (any day with contact with the healthcare system outside the home regardless of the duration or purpose) or a home day. If multiple healthcare encounters occurred on the same day (eg, outpatient labs, imaging, clinician visit), it was considered a single contact day but was counted within each source. We calculated overall percentage of contact as days contact days divided by overall survival.

**Fig. 1.**
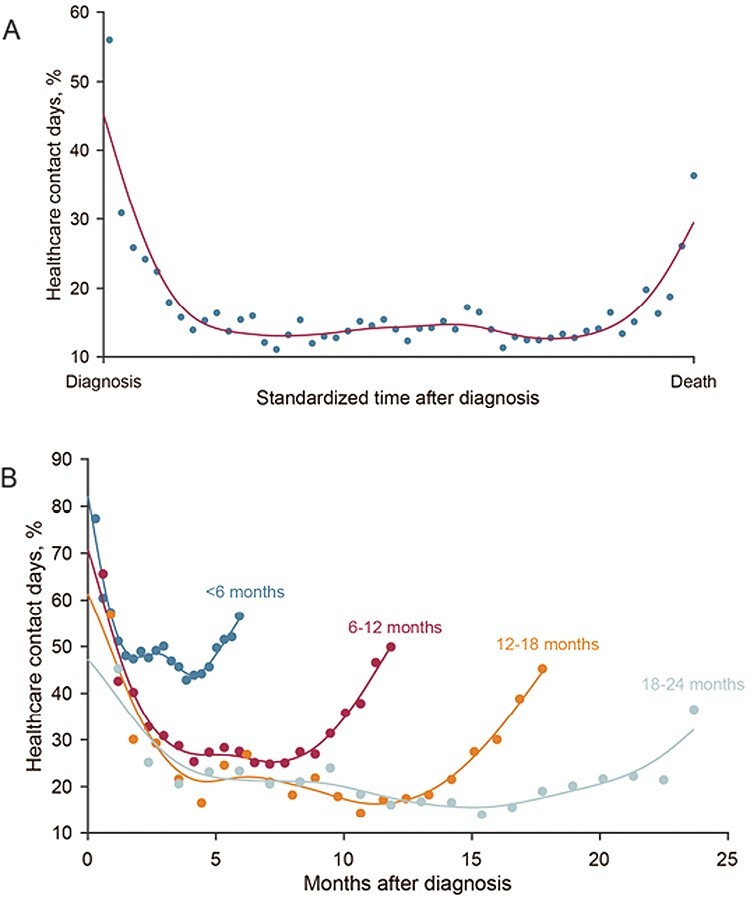
Percent of contact days from diagnosis to death for decedents with stage IV gastrointestinal malignancies for entire cohort (**A**), and grouped by duration of overall survival (**B**).

To demonstrate the trajectory of contact days over the course of illness, we standardized the time from diagnosis to death (x-axis) and calculated a cubic smoothing spline to plot percentage contact days over time (Fig. 1A). We hypothesized that trajectories could vary by survival and thus also plotted trajectories after grouping patients into survival cohorts (0-6 months, 6-12 months, etc.) (Fig. 1B).

Using multivariable beta regression, we analyzed clinicodemographic factors associated with percentage of contact days and reported these as adjusted odds ratios with 95% confidence intervals. The Minneapolis VA institutional review board reviewed the project and determined that it did not constitute human research.

## Results

We included 468 patients with stage IV gastrointestinal cancers. The most common primary sites were gastroesophageal (31.1%) and pancreatic (28.3%). The median age at diagnosis was 69 years (Table 1). Twenty percent of patients were 70% or more service-connected, and they were entitled to additional benefits due to medical conditions that were caused or worsened by military service.^7^

**Table 1.** Patient clinical factors and demographics.

Patient characteristic	Data
Age at diagnosis in years, median (IQR)	69 (64, 76)
Sex, Number (%)	
Male	463 (98.9)
Female	5 (1.1)
Charlson Score, median (IQR)	2 [1, 3]
70% or more service connected, number (%)	94 (20.0)
Race, number (%)	
White	387 (82.3)
Black or African American	28 (6.0)
Native Hawaiian or Other Pacific Islander	4 (0.9)
American Indian or Alaska Native	4 (0.9)
Other/Unknown	47 (10.0)
Ethnicity, number (%)	
Not Hispanic or Latino	448 (95.3)
Hispanic or Latino	3 (0.6)
Other/Unknown	19 (4.0)
Rurality, number (%)	
Urban	286 (60.9)
Rural	183 (38.9)
Unknown	1 (0.2)
Primary cancer location, number (%)	
Gastroesophageal	146 (31.1)
Pancreatic	133 (28.3)
Colorectal	120 (25.5)
Hepatobiliary	70 (14.9)
Other	1 (0.2)
Year of diagnosis, number (%)	
2010-2013	166 (35.3)
2014-2017	143 (30.4)
2018-2021	161 (34.3)
Receipt of any cancer-directed treatment, number (%)	218 (46.6)
Ever hospitalized from diagnosis to death, number (%)	413 (88.2)
Ever received care in emergency department, number (%)	306 (65.8)

The median (IQR) overall survival was 126 days (49-317). Less than half of patients (46.6%) received cancer-directed treatment. The median number and percentage of contact days were 44 days and 34.9%. Ambulatory visits were the most common source of contact days (61.4%). A majority (88.2%) of patients were hospitalized at least once, with a median 13 hospital days (Table 1, Supplementary Table S1).

The highest percentage of contact days occurred immediately after diagnosis (45%) and before death (32%) with a trough in the middle, forming a U-shaped curve (Fig. 1A). The “”U’’ was consistent across survival cohorts; the depth of the trough was deeper as survival increased (Fig. 1B).

Higher comorbidity index (aOR 1.09 [1.03-1.15]) and more recent diagnosis year (2014-2017, vs. 2010-2013, aOR 1.48 [1.1-1.99]) were associated with higher percentage of contact days. Conversely, rural residence (versus urban, OR 0.76 [0.59-0.97]) was associated with fewer percentage contact days (Supplementary Table S2).

## Discussion

In this single VA center report of decedents with stage IV gastrointestinal cancer, patients had a median survival of 4 months and spent 1 in 3 days with healthcare contact. Contact days followed a U-shaped trajectory and varied by clinical and sociodemographic factors.

The median survival of 4 months, while disappointing, is comparable to other contemporary real-world cohorts.^4^ The survival must be interpreted in the context of the underlying population, almost 60% with advanced gastroesophageal and pancreatic cancer (associated with worse prognosis), less than half received cancer-directed treatment (reflecting underlying sickness and ineligibility for treatment), and only included decedents (excluding longer-term survivors). The relatively lower rate of stage IV colorectal cancer in the cohort may relate to protocolized colorectal cancer screening at the VA, more coordinated preventive services at the VA are associated with improved cancer survival.^8^ Strikingly, patients spent one in every 3 days receiving predominantly outpatient healthcare, with a survival measured in months, similar to patients with advanced gastrointestinal cancer in non-VA settings.^4^

The percentage of contact days over time followed a “U-shaped” trajectory, with a higher proportion post-diagnosis and at the end-of-life. This pattern likely reflects the initial healthcare needs when patients present (eg, with jaundice), and the workup including staging and multidisciplinary input. As patients approach the end-of-life, healthcare needs rise again. Notably, the “”U’’ persisted when patients were divided into survival cohorts, but with deeper troughs the longer people lived. These results confirm the novel U-shaped contact days trajectory described recently in non-VA settings,^4^ also seen for health system costs. Interventions addressing the peaks or upswings of the U-curve (eg, multidisciplinary coordinated care and proactive palliative care) could reduce unnecessary contact days.^9^

Due to the unique population and care delivery within the VA, the findings of this study might have limited generalizability. However, the comprehensive care and robust data collection at the VA provide a unique opportunity to capture contact days. We did not include days with only telehealth visits or home-based care in the contact days measure, and this underestimates the total days with actual healthcare contact. Contact days also represent access to oftentimes necessary care, and care must be taken to not blindly interpret fewer contact days as universally good, especially for rural residents, who already face barriers to care.^10^ We only included decedents, which might exclude some long-term survivors. We did not judge the appropriateness of a contact day, and additional work is necessary to optimize judicious delivery of high-quality care.

## Supplementary Material

oyad313_suppl_Supplementary_Tables

## Data Availability

The data underlying this article will be shared on reasonable request to the corresponding author.

## Prior Presentation

This work was presented in abstract form at the 2023 ASCO Quality Care Symposium held in Boston, MA, USA on October 27-28, 2023
