# Supplementary material for: Trajectory of Healthcare Contact Days for Veterans With Advanced Gastrointestinal Malignancy
Source: Oncologist. 2023 Nov 28;29(2):e290–3. doi: 10.1093/oncolo/oyad313 (PMC10836304; doi:10.1093/oncolo/oyad313)
Supplement: oyad313_suppl_Supplementary_Tables [file oyad313_suppl_supplementary_tables.docx]

**Supplementary Tables:**

**Supplementary Table 1: Composition of healthcare contact day types among patients with stage IV gastrointestinal malignancies**

| **Time measure and/or source of contact days** | **Days,**  **median (IQR)** | **Percentage of contact days, median** |
| --- | --- | --- |
| Overall survival, diagnosis to death | 126 (49, 318) | NA |
| Healthcare contact days, total | 44 (20, 95) | 100.0% |
| Hospitalized days | 13 (5, 32) | 29.5% |
| Outpatient clinic visits | 27 (12, 61) | 61.4% |
| Labs | 17 (8, 40) | 38.6% |
| Radiology | 7 (4, 14) | 15.9% |
| Infusions | 6 (1, 17) | 13.6% |
| Emergency department | 1 (0, 3) | 2.3% |

**Supplementary Table 2: Beta regression analysis of patient sociodemographic or clinical factors associated with percentage of contact days for patients with stage IV gastrointestinal malignancies**

| **Characteristic** | **Adjusted odds ratio (95% CI)** | **P value** |
| --- | --- | --- |
| Age |  |  |
| Less than 55 years | 1 [Reference] | N/A |
| 55-64 years | 0.83 (0.46-1.5) | 0.55 |
| 65-74 years | 0.73 (0.41-1.29) | 0.28 |
| More than 75 years | 0.64 (0.35-1.17) | 0.15 |
| Charlson Score | 1.09 (1.03-1.15) | <0.01 |
| Service Connected, % |  |  |
| <70% | 1 [Reference] | N/A |
| >= 70% | 1.02 (0.77-1.35) | 0.89 |
| Race |  |  |
| White | 1 [Reference] | N/A |
| American Indian or Alaska Native | 5.65 (1.78-17.93) | <0.01 |
| Other/Unknown | 1.38 (0.9-2.13) | 0.14 |
| Black or African American | 0.9 (0.55-1.49) | 0.69 |
| Native Hawaiian or Other Pacific Islander | 0.37 (0.1-1.37) | 0.14 |
| Ethnicity |  |  |
| Not Hispanic | 1 [Reference] | N/A |
| Hispanic | 0.41 (0.1-1.72) | 0.23 |
| Unknown | 0.69 (0.34-1.4) | 0.3 |
| Rurality |  |  |
| Urban | 1 [Reference] | N/A |
| Rural | 0.76 (0.59-0.97) | 0.03 |
| Unknown | 0.24 (0.02-3.17) | 0.28 |
| Primary Site |  |  |
| Colon | 1 [Reference] | N/A |
| Esophagus | 1.12 (0.79-1.59) | 0.53 |
| Gallbladder | 1 (0.38-2.62) | 1 |
| Liver | 0.72 (0.48-1.07) | 0.11 |
| Pancreas | 1.18 (0.84-1.66) | 0.34 |
| Rectum | 1.3 (0.77-2.19) | 0.33 |
| Stomach | 1.07 (0.7-1.63) | 0.76 |
| Other | 8.1 (0.86-76.07) | 0.07 |
| Year of Diagnosis |  |  |
| 2010-2013 | 1 [Reference] | N/A |
| 2014-2017 | 1.48 (1.1-1.99) | 0.01 |
| 2018-2021 | 1.53 (1.13-2.08) | 0.01 |
| Receipt of any cancer-directed treatment |  |  |
| No | 1 [Reference] | N/A |
| Yes | 0.36 (0.28-0.46) | <0.01 |

In beta regression analysis, patients with a higher Charlson comorbidity index were more likely to experience a higher percentage of healthcare contact days. Patients living in rural areas had fewer contact days compared to their urban counterparts, potentially indicating replacement of in-person care with telehealth services (which were not included as contact days in this study). Fewer contact days could also reflect challenges patients residing in rural areas face in accessing oncology services. Diagnosis year after 2013 was also associated with more contact days — this could represent the growing complexity of oncology care over time, or a selection bias with patients diagnosed later having overall shorter survival and potentially poorer health status. Receipt of cancer-directed treatment was associated with a lower percentage of contact days— this likely reflects the longer survival of this population— and the chance to experience the lower middle trough— and does not imply how cancer treatment itself impacts contact days. Patients who receive supportive care alone have persistently high healthcare needs and short survival, and thus a higher percentage of contact days. These results do not imply that receipt of cancer-directed therapy decreases contact days.
